# Validation of normal reference ranges in cardiac magnetic resonance imaging: The Multi-Ethnic Study of Atherosclerosis

**DOI:** 10.1016/j.jocmr.2025.101949

**Published:** 2025-08-26

**Authors:** Nadine Kawel-Boehm, Spencer L. Hansen, Bharath Ambale-Venkatesh, J. Jeffrey Carr, J. Paul Finn, Michael Jerosch-Herold, Steven M. Kawut, Robyn L. McClelland, Wendy Post, Martin R. Prince, Steven Shea, João A.C. Lima, David A. Bluemke

**Affiliations:** a Department of Radiology, Kantonsspital Graubuenden, Chur, Switzerland; b Department of Biostatistics, University of Washington, Washington, Washington, USA; c Department of Radiology, Johns Hopkins University, Baltimore, Maryland, USA; d Department of Radiology, Vanderbilt University Medical Center, Nashville, Tennessee, USA; e Department of Radiology, University of California, Los Angeles, California, USA; f Department of Radiology, Brigham and Women’s Hospital, Boston, Massachusetts, USA; g Pulmonary, Allergy, and Critical Care Division, University of Pennsylvania, Philadelphia, Pennsylvania, USA; h Center for Clinical Epidemiology and Biostatistics, Allergy, and Critical Care Division, University of Pennsylvania, Philadelphia, Pennsylvania, USA; i Department of Epidemiology, Johns Hopkins University, Baltimore, Maryland, USA; j Department of Radiology, Cornell and Columbia Universities, New York, New York, USA; k Departments of Medicine and Epidemiology, Columbia University, New York, New York, USA; l Division of Cardiology, Johns Hopkins University, Baltimore, Maryland, USA; m Department of Radiology, University of Wisconsin, Madison, Wisconsin, USA

**Keywords:** Normal values, Reference range, Cardiovascular magnetic resonance, Cardiovascular events

## Abstract

**Background:**

Normal reference ranges in cardiovascular imaging studies are typically established as the mean value plus and minus twice the standard deviation (SD) of a healthy reference cohort (“2 SD-method”). Although widely used for cardiac magnetic resonance (CMR), this approach has not been previously validated. The purpose of this study was to use longitudinal cohort data to assess the clinical predictive validity of normal reference values for cardiac CMR.

**Methods:**

Normal reference ranges for left- and right ventricular (LV and RV) CMR parameters were derived from baseline exam data of 1518 participants (age 45–84 years) in the Multi-Ethnic Study of Atherosclerosis (MESA) study without known CV disease and without established CV risk factors. Cut-off values at 1 and 2 SDs were obtained for the following LV and RV parameters indexed to body surface area: end-diastolic volume (LVEDVi, RVEDVi), end-systolic volume (LVESVi, RVESVi), mass (LVMi, RVMi), as well as for LVED diameter (LVEDD), LVED wall thickness, and ejection fraction (LVEF, RVEF). The relationship of reference values to CV events was then evaluated in the entire MESA cohort with CMR data (*n *= 4915), including individuals with CV risk factors at the baseline exam. Cox proportional hazard models were calculated for major adverse and all CV events (MACE and ACE, respectively) at 5 and 10 years of follow-up.

**Results:**

At 5 years of follow-up, LVEDVi, LVESVi, and LVEF beyond the 2SD-threshold of the mean reference values were predictors of MACE and ACE in men and women (HR 2.1–4.3; *P*<.001–.029). In men, LVMi and LVED wall thickness above the 1 SD-threshold were associated with CV events (HR 1.6–2.1; *P*<.001–.002). For women, LVED wall thickness above the 1 SD-threshold significantly increased risk of adverse events (HR 1.6–2.3; *P*.034–.002) while LVMi was associated with events only for values above the 2SD-threshold (HR 2.7–4.1; *P*<.001). Notably, LVEDD, RVMi, RVESVi, and RVEF were not associated with CV events in men or women. CV events over 10 years showed similar trends.

**Conclusion:**

Our results support the clinical relevance of CMR normal reference ranges for LV parameters. Most LV CMR parameters beyond the normal reference range (2SD-threshold) were associated with elevated CV risk at 5 and 10 years. Elevated LVEDDi, RVMi, RVESVi, and RVEF, however, were not associated with CV events.

## Graphical abstract

**Figure:**
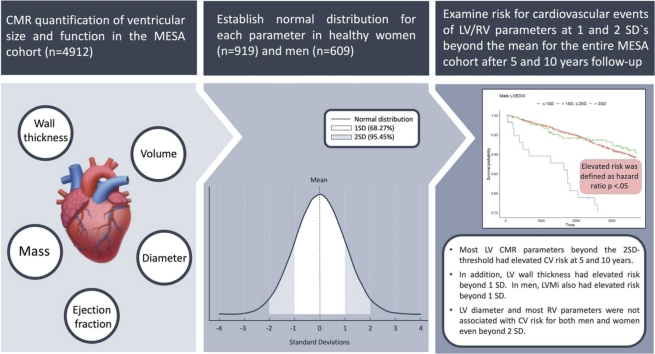

## 1. Introduction

Cardiovascular magnetic resonance (CMR) enables quantification of various cardiovascular (CV) parameters related to myocardial structure and function. CMR is widely accepted as the clinical reference standard for heart dimensions and function [1]. To distinguish between “normal” and “abnormal” measurements, normal reference ranges have been established in healthy reference populations [2]. Individuals with CMR parameters beyond these normal values are commonly flagged as “abnormal” in CMR reports.

By convention for both CMR and echocardiography, upper and lower limits of a reference range are typically delineated as the mean value minus and plus twice the standard deviation of a parameter (“2 SD-method”). Statistically, this definition results in approximately 95% of the data points within a normal distribution being within the limit. This arbitrary choice of reference ranges is in line with common usage for clinically assessed anthropometric, serologic, and imaging markers and beyond scientific and actuarial value aims to convey a measure of personal risk relative to the majority of individuals in a given population. Current CMR guidelines advise using reference intervals for reporting [3]. However, to our knowledge, the clinical relevance of reference ranges for many CMR parameters has not been previously evaluated.

Most cardiac parameters show variation by sex and body size [2], [4]. Therefore, in order to better predict risk, separate reference ranges have been established for men versus women, and further adjusted for body surface area (BSA). Previous work has shown that other, more complex allometric measures result in better adjustment for body size than BSA, although differences are generally small or null, in terms of risk prediction [5].

The purpose of this study was to assess the clinical significance of established ranges of normal reference values for CMR. We evaluated men and women with no known clinical CV disease at baseline. A subset of these individuals without traditional CV risk factors (defined as “normal individuals”) was used to derive population means for left ventricular (LV) and right ventricular (RV) parameters. The clinical significance of cut-off values at various thresholds for CMR parameters above the mean values for a multi-ethnic population of asymptomatic individuals free of traditional cardiovascular risk factors was then calculated based on the subsequent occurrence of CV events at 5- and 10-year follow-up.

## 2. Materials and methods

### 2.1. Study sample

The Mult-Ethnic Study of Athersclerosis (MESA) is a community-based longitudinal cohort study (*ClinicalTrials.gov*: NCT00005487) initiated in July 2000 that has been described in detail previously [6]. In brief, 6814 participants (aged 45–84 years) of four different race/ethnicities (White, Black, Hispanic, Chinese), free of clinically recognized cardiovascular disease at baseline, were recruited from six U.S. communities (Baltimore City and Baltimore County, Maryland; Chicago, Illinois; Forsyth County, North Carolina; Los Angeles County, California; Northern Manhattan and the Bronx, New York City, New York; and St. Paul, Minnesota). To determine parameters of cardiac size and function, 4999 participants also underwent a CMR exam.

The MESA study was approved by the institutional review boards of each of the participating field sites in the United States (Wake Forest University, Winston-Salem, North Carolina; Columbia University, New York, New York; Johns Hopkins University, Baltimore, Maryland; University of Minnesota, Minneapolis, Minnesota; Northwestern University, Evanston, Illinois; and University of California, Los Angeles, California), and all participants provided written informed consent. All sites were compliant with the Health Insurance Portability and Accountability Act.

### 2.2. Risk factor measures

MESA participants underwent an extensive evaluation, including clinical history, physical examination, laboratory tests, and anthropometric measurements. Standard questionnaires were used to obtain information regarding demographics, smoking history defined as current, former, or never, current medication including hypoglycemic, and antihypertensive drugs, and physician diagnosis of hypertension and diabetes.

Weight was measured to the nearest 0.5 kg and height to the nearest 0.1 cm. Body surface area (BSA) was calculated as 0.20247 × [height (m) ^(0.725)^] x [weight (kg) ^(0.425)^] and body mass index was calculated as weight in kilogram divided by height in meters squared. Resting blood pressure was measured three times in seated participants; the average of the last two measurements was used in the analysis. Total and high-density lipoprotein cholesterol and glucose levels were measured from blood samples obtained after a 12-h fast.

### 2.3. Image acquisition and assessment

CMR parameters of LV and RV size and systolic function were assessed using 1.5 Tesla scanners (Avanto and Espree, Siemens Healthineers, Erlangen, Germany), and Signa HD, General Electric, Waukesha, Wisconsin) as described previously [7]. In brief, a stack of short-axis, non-contiguous slices covering the entire LV and RV was acquired using a cine fast gradient echo sequence with temporal resolution less than or equal to 40 msec. Contouring of the LV and RV endocardial and epicardial borders was performed by means of a semiautomated software (Q-MASS 4.2 Medis, Leiden, The Netherlands) with manual correction. LV and RV end-diastolic and LV and RV end-systolic volumes were calculated by summation of the areas on each slice multiplied by the sum of the slice thickness and image gap (Simpson’s rule). LV and RV mass were calculated at end-diastole as the sum of the myocardial area (area between endocardial and epicardial contours) times the slice thickness plus the interslice gap multiplied by the specific gravity of myocardium (1.05 g/mL). LV end-diastolic diameter was measured as the average internal diameter on midventricular short-axis images. LV end-diastolic wall thickness was calculated as the average diameter of the six segments at midventricular short-axis.

### 2.4. Assessment of cardiovascular events

Adjudication of cardiovascular events in MESA has been published in detail previously [8] and is briefly summarized here. To obtain data regarding hospital admissions, CV outpatient diagnosis, and deaths, MESA participants were followed with five in-person study examinations and telephone interviews every 9–12 months after baseline examination. Copies of death certificates and medical records of hospitalizations were requested to ascertain cardiovascular events and mortality. Two independent physicians from the MESA study events committee classified the end-point according to pre-specified MESA study criteria, blinded to all participant data. In cases of disagreement, final decision was made by the entire events committee.

The composite end-point of major adverse cardiovascular events (MACE) was defined as myocardial infarction (MI), resuscitated cardiac arrest, stroke, heart failure, and mortality related to coronary heart disease (CHD), CV disease (CVD), arteriosclerotic disease, or stroke. The composite end-point of all cardiovascular events (ACE) was defined as MACE, angina, peripheral vascular disease (PVD), percutaneous transluminal coronary angioplasty (PTCA), coronary artery bypass grafting (CABG), and transient ischemic attack (TIA).

Diagnosis of MI was based on clinical criteria (chest pain), ECG criteria, and cardiac biomarker levels as described previously [8]. Mortality related to CHD included in-hospital death due to MI, out-of-hospital death with a documented MI within 28 days, chest pain within 72 h before death, or a history of CHD, and the absence of a known non-atherosclerotic or non-cardiac cause of death. Criteria for heart failure (HF) included a diagnosis of HF by a physician and medical treatment for HF (probable HF) or definite HF which also required pulmonary edema by radiography or dilated ventricle or poor LV function or evidence of LV diastolic dysfunction.

### 2.5. Statistical analysis

Descriptive statistics are presented as means and standard deviations for the continuous parameters and percent and number of participants for the discrete parameters (Table 1). Normal reference ranges of BSA-adjusted quantitative CMR parameters of ventricular volumes (LVEDVi, RVEDVi, LVESVi, and RVESVi), mass (LVMi and RVMi), and ejection fraction (LVEF and RVEF) as well as LV end-diastolic diameter (LVEDD) and end-diastolic wall thickness (LVED wall thickness) were calculated for a “healthy” subset of male and female MESA participants, without known CV disease at baseline enrollment and without traditional CV risk factors (including hypertension, diabetes, smoking, and elevated cholesterol). CV risk factors were specified as systolic blood pressure >140 mm Hg, diastolic blood pressure >90 mmHg, use of antihypertensive medication, fasting blood glucose >110 mg/dl, diabetes medication use, total cholesterol >240 mg/dl, high-density lipoprotein (HDL) cholesterol <40 mg/dl, and current smoking.

**Table 1:** Baseline characteristics of the MESA study cohort for analysis of LV parameters.

Characteristic	Entire cohort (n = 4912)	Participants without CV risk factors (n = 1528)	Participants with CV risk factors (n = 3384)
Age, mean (SD), y	61.5 (10.1)	58.8 (9.9)	62.7 (10.0)
Sex, No. (%)			
Women	2575 (52%)	919 (60%)	1656 (49%)
Men	2337 (48%)	609 (40%)	1728 (51%)
Race/ethnicity, No. (%)			
White	1919 (39%)	697 (46%)	1222 (36%)
Chinese	647 (13%)	262 (17%)	385 (11%)
Black/ African American	1253 (26%)	274 (18%)	979 (29%)
Hispanic	1093 (22%)	295 (19%)	798 (24%)
Height, mean (SD), m	1.7 (0.1)	1.7 (0.1)	1.7 (0.1)
Weight, mean (SD), kg	77.0 (16.1)	72.8 (15.5)	78.9 (16.0)
Body mass index, mean (SD), kg/m^2^	27.7 (4.9)	26.3 (4.6)	28.4 (4.9)
Body surface area, mean (SD), m^2^	1.8 (0.2)	1.8 (0.2)	1.9 (0.2)
Cholesterol, mean (SD), mg/dL			
Total	194 (35)	191 (26)	196 (39)
HDL	51 (15)	57 (14)	49 (15)
Fasting glucose, mean (SD), mg/dl	96 (29)	86 (9)	101 (34)
Diabetic medication use, No. (%)			
No	4469 (91%)	1528 (100%)	2941 (87%)
Yes	443 (9%)	0 (0%)	443 (13%)
Blood pressure, mean (SD), mmHg			
Systolic	125 (21)	114 (13)	131 (22)
Diastolic	72 (10)	68 (9)	73 (11)
Hypertension medication use, No. (%)			
No	3178 (65%)	1528 (100%)	1650 (49%)
Yes	1734 (35%)	0 (0%)	1734 (51%)
Smoking status, No. (%)			
Never	2528 (51%)	937 (61%)	1591 (47%)
Former smoker	1760 (36%)	591 (39%)	1169 (35%)
Current smoker	624 (13%)	0 (0%)	624 (18%)

*CV* cardiovascular, *SD* 1 standard deviation, *HDL* high-density lipoprotein

According to histograms, all CMR variables were normally distributed. In accordance with common practice and previous publications, the lower limit of the reference range was calculated as the mean value minus twice the standard deviation and the upper limit as the mean value plus twice the standard deviation (2SD-thresholds) [2], [9]. Additionally, we calculated the thresholds at the mean ± 1 SD (1SD-threshold). Normal reference ranges at 3 SDs from the mean were also explored, but there were insufficient CV events at those cut-off values.

Risk prediction of the different SD-thresholds were tested on the entire MESA cohort, including “healthy” participants and participants with CV risk factors, by calculating unadjusted Cox proportional hazard models for both endpoints (MACE and ACE) by comparing three groups of quantitative CMR parameters in separate analysis for men and women: for parameters where the upper limit is clinically more relevant (LVMi, RVMi, LVEDVi, RVEDVi, LVESVi, RVESVi, LVEDD, and LVED wall thickness) the three groups consisted of participants with values below or equal to the 1 SD-threshold (≤mean + 1*SD) versus participants with values above the 1 SD-threshold and below or equal to the 2 SD-threshold (>mean + 1*SD and ≤mean + 2*SD) versus subjects with a value above the 2 SD-threshold (>mean + 2*SD). For EF, where the lower limit is clinically more relevant, participants with values ≥ mean - 1*D were compared to participants with values < mean - 1*SD and ≥ mean - 2*SD, and subjects with values < mean - 2*SD, respectively. Statistical significance was determined by a *P*-value <.05. Similar to clinical reporting standards, we did not calculate models adjusted for CV risk factors.

Kaplan-Meier curves were generated to display occurrence of events over time according to baseline values of each parameter, comparing the three groups based on different SD-thresholds.

All analyses were performed with R software version 4.4.2.

## 3. Results

### 3.1. Study population

**Fig. 1:**
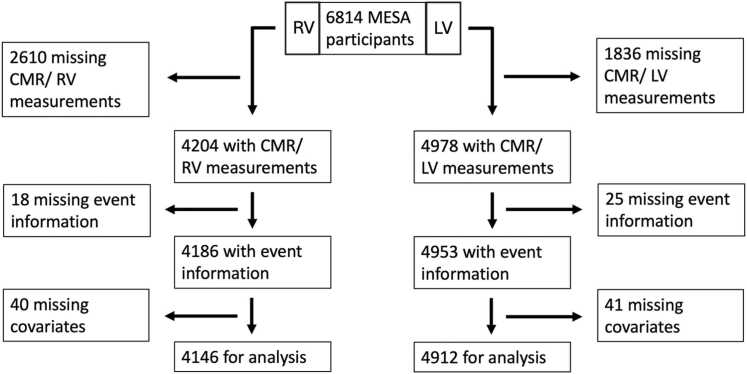
Study participant flow chart. *MESA* multi-ethnic study in atherosclerosis, *CMR* cardiovascular magnetic resonance, *LV* left ventricle, *RV* right ventricle

Of 6814 MESA participants, 4912 (2575 women and 2337 men) completed a CMR exam and had complete data on parameters of LV size and function, follow-up events, demographics, and risk factors (Fig. 1). For the analysis of RV parameters data of 2178 women and 1968 men were available. The mean age of participants at baseline was 62 years (for analysis of LV parameters) and 61 years (for analysis of RV parameters), respectively. 39% of the participants were White, 13% Chinese, 26% of African American and 22% of Hispanic origin. 36% (919/2575) [35% (752/2178)] of female and 26% (609/2337) [26% (505/1968)] of male participants included in the analysis of LV and RV parameters, respectively, were free of known CV disease at baseline and did not have CV risk factors.

Table 1 shows baseline characteristics for the entire cohort of the LV analysis, for participants without known CV disease and risk factors and for participants with cardiovascular risk factors. Supplemental Table 1 shows baseline characteristics of the cohort for RV analysis.

### 3.2. Cardiovascular events

After 5 and 10 years of follow-up, 3.1% and 6.3% of women, respectively, developed MACE. For men after 5 and 10 years of follow-up, 5.2% and 10.7%, respectively, developed MACE. Event rates for ACE at 5 and 10 years were slightly higher (Table 2). Cardiovascular events that occurred in participants available for the analysis of RV parameters are shown in Table 2. Event counts of the individual components of MACE and ACE are listed in Supplemental Table 2.

**Table 2:** Number of cardiovascular events.

Ventricle	Sex	N	5-year MACE	10-year MACE	5-year ACE	10-year ACE
LV	Female	2575	80	161	127	247
	Male	2337	122	250	226	396
RV	Female	2178	72	146	116	223
	Male	1968	101	197	193	326

*MACE* major adverse cardiovascular events, *MI* myocardial infarction, *CHD* resuscitated cardiac arrest, stroke, mortality related to coronary heart disease, *CVD* cardiovascular disease, arteriosclerotic disease or stroke, heart failure, *ACE* all cardiovascular events, *PVD* peripheral vascular disease, *PTCA* percutaneous transluminal coronary angioplasty, *CABG* coronary artery bypass grafting, *TIA* transient ischemic attack; *LV* left ventricle; *RV* right ventricle

### 3.3. Standard deviation thresholds

Mean values and 1- and 2-standard deviation thresholds for CMR-derived variables of LV and RV size and function were calculated based on the 919 (LV) and 752 (RV), respectively, female and the 609 (LV) and 505 (RV), respectively, male participants free of known CV disease and risk factors (Supplemental Table 3).

When mean values and SD thresholds were applied to the entire cohort of 2575 female participants available for analysis of LV parameters (including those with and without CV risk factors), between 10% and 19% of female participants had LV structure or function values between the 1SD- and 2SD-threshold (depending on the variable); between 3% and 10% of women had measurements beyond the 2SD-threshold per variable. Similarly, between 8% and 17% of 2337 male participants had values between the 1SD- and 2SD-threshold, and 2% to 8% had measurements beyond the 2SD-threshold.

Regarding RV parameters, between 10% and 12% of 2178 female participants had values between the 1SD- and 2SD-threshold, and between 2% and 4% of women had measurements beyond the 2SD-threshold per variable. Around 9–10% of 1968 male participants had values between the 1SD- and 2SD-threshold and 2% had measurements beyond the 2 SD-threshold for each RV-parameter. The number and percentages of female and male participants of the entire cohort beyond the 1- and 2-SD cut-offs are listed in Table 3 for each variable.

**Table 3:** Participants [n, (%)] beyond the 1- and 2-standard deviation-thresholds, respectively.

	Female (n = 2575)		Male (n = 2337)	
Variable	+ 1 SD	+ 2 SD	+ 1 SD	+ 2 SD
LVMi (g/m^2^)	461 (18)	248 (10)	331 (14)	148 (6)
LVEDVi (ml/m^2^)	279 (11)	82 (3)	209 (9)	59 (3)
LVESVi (ml/m^2^)	262 (10)	91 (4)	196 (8)	87 (4)
LVEDD (mm/m^2^)	277 (11)	65 (3)	254 (11)	41 (2)
LVED wall thickness (mm)	481 (19)	235 (9)	388 (17)	176 (8)
RVMi (g/m^2^)	435 (11)	57 (3)	180 (9)	42 (2)
RVEDVi (ml/m^2^)	253 (12)	50 (2)	180 (9)	38 (2)
RVESVi (ml/m^2^)	227 (10)	77 (4)	180 (9)	47 (2)
Variable	- 1 SD^1^	- 2 SD^1^	- 1 SD^1^	- 2 SD^1^
LVEF (%)	287 (11)	83 (3)	274 (12)	94 (4)
RVEF (%)	242 (11)	82 (4)	224 (11)	53 (3)

*LV* left ventricular, *M* mass, *i* indexed to body surface area, *EDV* end-diastolic volume, *ESV* end-systolic volume, *EDD* end-diastolic diameter, *RV* right ventricular, *EF* ejection fraction, *SD* standard deviation

+ 1 SD = participants with values > mean + 1*SD and ≤ mean + 2*SD

+ 2 SD = participants with a value > mean + 2*SD (upper limit of the normal reference range) - 1 SD = participants with values < mean - 1*SD and ≥ mean - 2*SD

- 2 SD = participants with values < mean - 2*SD (lower limit of the normal reference range)

### 3.4. Relationship of CMR variables to MACE and ACE

**Fig. 2:**
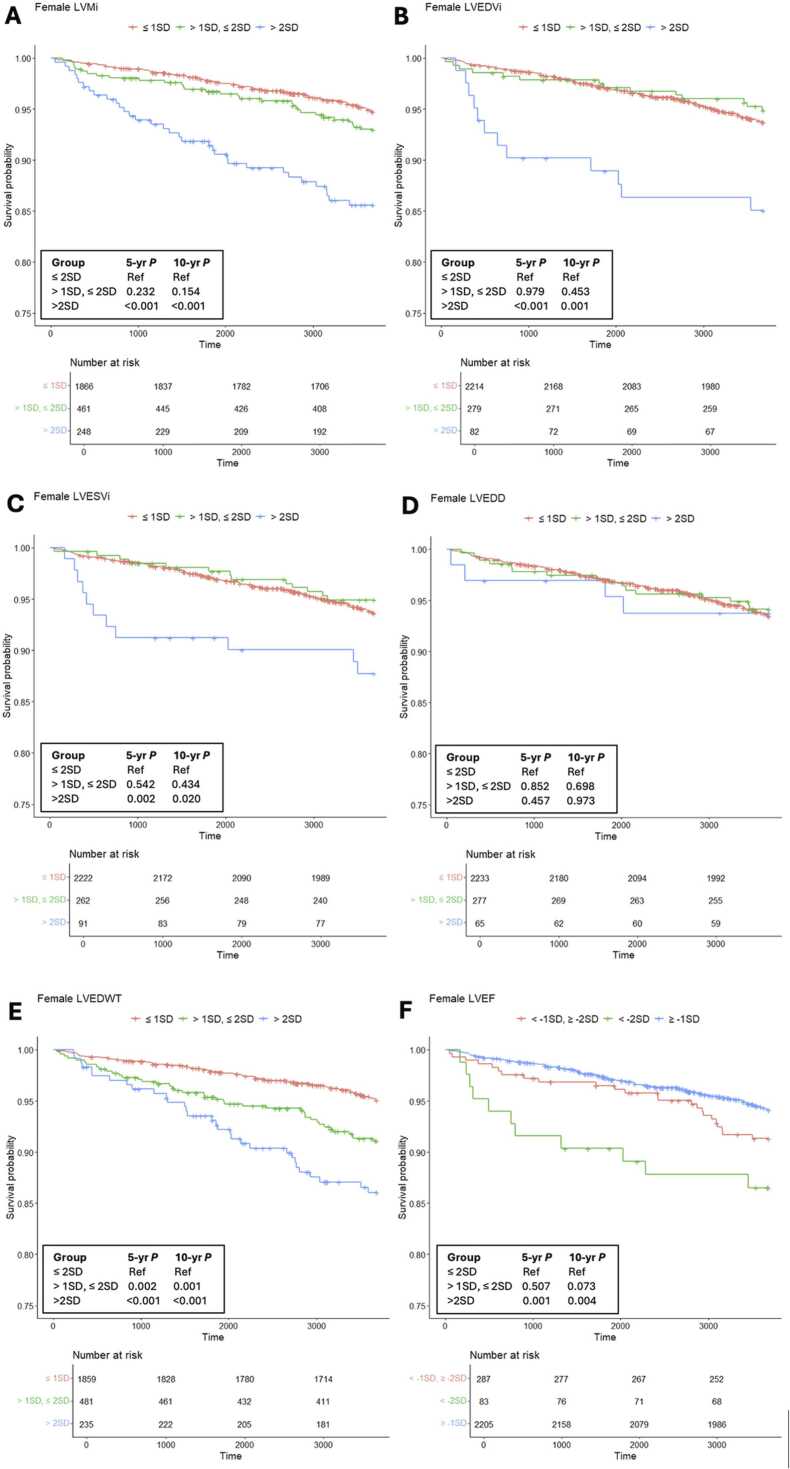
Kaplan-Meier curves of survival free from MACE of all female participants for LVMi **(A)**, LVEDVi **(B)**, LVESVi **(C)**, LVEDD **(D)**, LVEDWT **(E)** and LVEF **(F)** comparing three groups: participants with values ≤ mean + 1*SD **(red line in A, B, C, D, E)** versus participants with values > mean + 1*SD and ≤ mean + 2*SD **(green line in A, B, C, D, E)** versus participants with a value > mean + 2*SD = upper limit of the normal reference range) **(blue line in A, B, C, D, E)** and participants with values ≥ mean - 1*SD **(blue line in F)** versus participants with values < mean - 1*SD and ≥ mean - 2*SD **(red line in F)** versus participants with values < mean - 2*SD = lower limit of the normal reference range **(green line in F)**. *MACE* major adverse cardiovascular events, *LVMi* left ventricular mass indexed to body surface area, *LVEDVi* left ventricular end-diastolic volume indexed to body surface area, *LVEDD* left ventricular end-systolic volume indexed to body surface area, *LVEDWT* left ventricular end-diastolic wall thickness, *LVEF* left ventricular ejection fraction

**Fig. 3:**
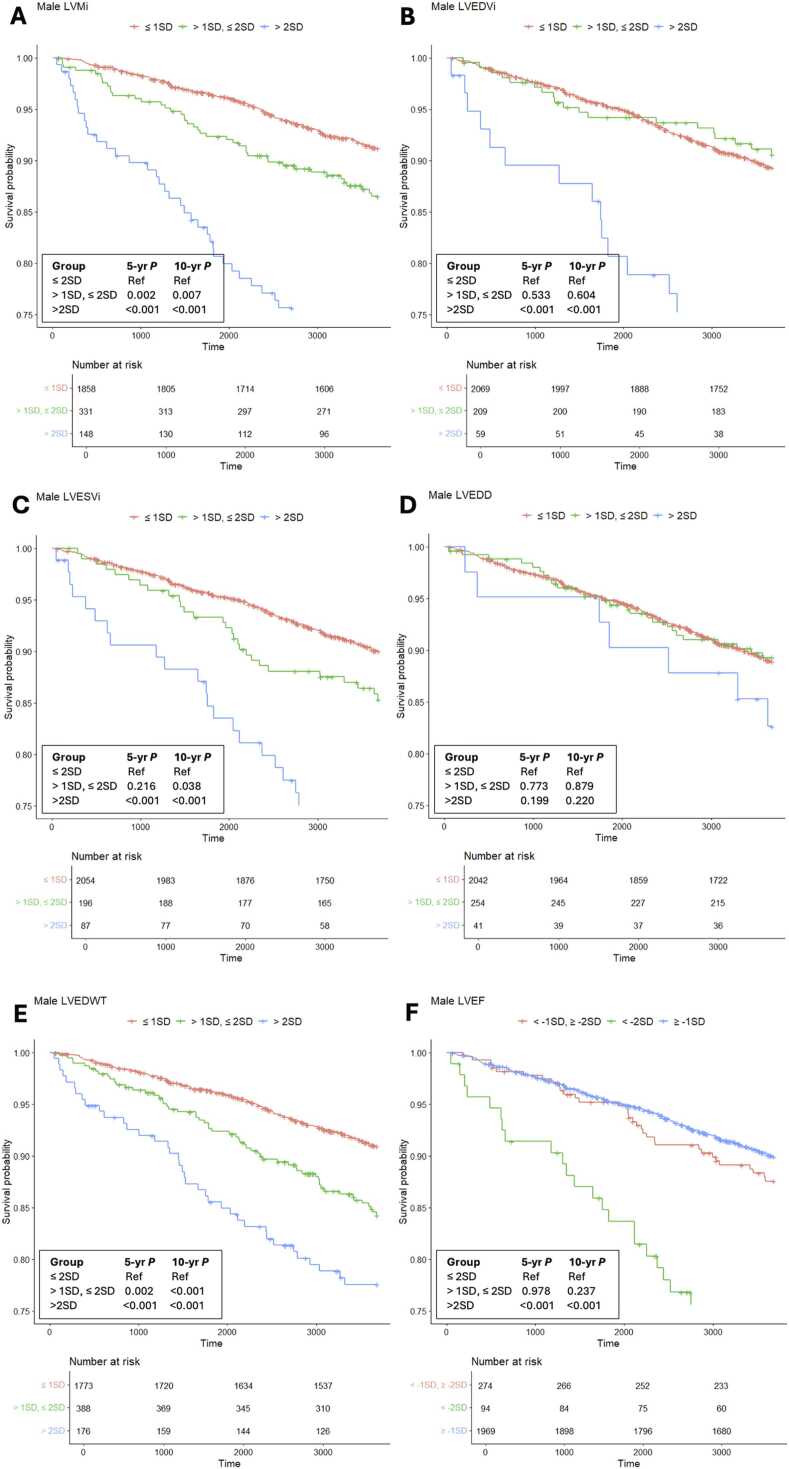
Kaplan-Meier curves of survival free from MACE of all male participants for LVMi **(A)**, LVEDVi **(B)**, LVESVi **(C)**, LVEDD **(D)**, LVEDWT **(E)** and LVEF **(F)** comparing three groups: participants with values ≤ mean + 1*SD **(red line in A, B, C, D, E)** versus participants with values > mean + 1*SD and ≤ mean + 2*SD **(green line in A, B, C, D, E)** versus participants with a value > mean + 2*SD = upper limit of the normal reference range) **(blue line in A, B, C, D, E)** and participants with values ≥ mean - 1*SD **(blue line in F)** versus participants with values < mean - 1*SD and ≥ mean - 2*SD **(red line in F)** versus participants with values < mean - 2*SD = lower limit of the normal reference range **(green line in F)**. MACE major adverse cardiovascular events, *LVMi* left ventricular mass indexed to body surface area, *LVEDVi* left ventricular end-diastolic volume indexed to body surface area, *LVEDD* left ventricular end-systolic volume indexed to body surface area, *LVEDWT* left ventricular end-diastolic wall thickness, *LVEF* left ventricular ejection fraction, *SD* standard deviation

In the analysis below, we sought to determine if CMR LV and RV structure and function parameters at various standard deviation cut-offs beyond mean values were related to MACE and ACE. Cox proportional hazards models for a follow-up of 5 years and 10 years were assessed for statistical significance (Table 4 **and** Supplemental Table 4). Kaplan-Meier curves were used to display event rates for men and women for each of the endpoints (Fig. 2, Fig. 3).

**Table 4:** Relationship of left ventricular CMR variables to cardiovascular events at 5-year follow-up assessed by the Cox proportional hazards model (unadjusted).

	Female (n = 2575)						Male (n = 2337)					
	MACE			ACE			MACE			ACE		
Variable Group^a^	Events *n* (%)^b^	HR (95% CI)	*P*	Events *n* (%)^b^	HR (95% CI)	*P*	Events *n* (%)^b^	HR (95% CI)	*P*	Events *n* (%)^b^	HR (95% CI)	*P*
LVMi												
≤1SD	43 (2)	Reference		77 (4)	Reference		69 (7)	Reference		144 (8)	Reference	
>1 SD, ≤2SD	15 (3)	1.4 (0.8−2.6)	0.232	24 (5)	1.3 (0.8−2.0)	0.297	25 (8)	2.1 (1.3−3.3)	**0.002**	45 (14)	1.8 (1.3−2.5)	**<0.001**
>2 SD	22 (9)	4.1 (2.4−6.8)	**<0.001**	26 (11)	2.7 (1.7−4.2)	**<0.001**	28 (19)	5.6 (3.6−8.8)	**<0.001**	37 (25)	3.6 (2.5−5.2)	**<0.001**
LVEDVi												
≤1SD	63 (3)	Reference		106 (5)	Reference		99 (5)	Reference		191 (9)	Reference	
>1 SD, ≤2SD	8 (3)	1.0 (0.5−2.1)	0.979	11 (4)	0.8 (0.4−1.5)	0.520	12 (6)	1.2 (0.7−2.2)	0.533	21 (10)	1.1 (0.7−1.7)	0.697
>2 SD	9 (11)	4.1 (2.1−8.3)	**<0.001**	10 (12)	2.7 (1.4−5.2)	**0.002**	11 (19)	4.3 (2.3−8.1)	**<0.001**	14 (24)	3.0 (1.7−5.1)	**<0.001**
LVESVi												
≤1SD	66 (3)	Reference		107 (5)	Reference		95 (5)	Reference		185 (9)	Reference	
>1 SD, ≤2SD	6 (2)	0.8 (0.3−1.8)	0.542	11 (4)	0.9 (0.5−1.6)	0.664	13 (7)	1.4 (0.8−2.6)	0.216	17 (9)	1.0 (0.6−1.6)	0.868
>2 SD	8 (9)	3.1 (1.5−6.5)	**0.002**	9 (10)	2.1 (1.1−4.2)	**0.029**	14 (16)	3.8 (2.2−6.6)	**<0.001**	24 (28)	3.5 (2.3−5.4)	**<0.001**
LVEDD												
≤1SD	68 (3)	Reference		106 (5)	Reference		104 (5)	Reference		195 (10)	Reference	
>1 SD, ≤2SD	9 (3)	1.1 (0.5−2.1)	0.852	17 (6)	1.3 (0.8−2.2)	0.328	14 (6)	1.1 (0.6−1.9)	0.773	27 (11)	1.1 (0.7−1.7)	0.598
>2 SD	3 (5)	1.1 (0.5−4.9)	0.457	4 (6)	1.3 (0.5−3.6)	0.591	4 (10)	1.9 (0.7−5.2)	0.199	4 (10)	1.0 (0.4−2.7)	0.989
LVEDWT												
≤1SD	40 (2)	Reference		75 (4)	Reference		68 (4)	Reference		145 (8)	Reference	
>1 SD, ≤2 SD	23 (5)	2.3 (1.4−3.8)	**0.002**	30 (6)	1.6 (1.0−2.4)	**0.034**	29 (8)	2.0 (1.3−3.1)	**0.002**	49 (13)	1.6 (1.2−2.2)	**0.004**
>2 SD	17 (7)	3.5 (2.0−6.1)	**<0.001**	22 (9)	2.5 (1.5−3.9)	**<0.001**	25 (14)	4.0 (2.5−6.3)	**<0.001**	32 (18)	2.4 (1.5−3.5)	**<0.001**
LVEF												
≥−1SD	62 (3)	Reference		104 (5)	Reference		94 (5)	Reference		180 (9)	Reference	
<−1SD, ≥−2SD	10 (4)	1.3 (0.6−2.4)	0.507	14 (5)	1.0 (0.6−1.8)	0.864	13 (5)	1.0 (0.6−1.8)	0.978	22 (8)	0.9 (0.6−1.4)	0.568
<−2SD	8 (10)	3.6 (1.7−7.5)	**0.001**	9 (11)	2.4 (1.2−4.8)	**0.011**	15 (16)	3.6 (2.1−6.2)	**<0.001**	24 (26)	3.2 (2.1−5.0)	**<0.001**

*MACE* major adverse cardiovascular events, *MI* myocardial infarction, *CHD* resuscitated cardiac arrest, stroke, mortality related to coronary heart disease, *CVD* cardiovascular disease, arteriosclerotic disease or stroke, heart failure, *ACE* all cardiovascular events,MACE, angina, *PVD* peripheral vascular disease, *PTCA* percutaneous transluminal coronary angioplasty, *CABG* coronary artery bypass grafting, *TIA* transient ischemic attack, *HR* hazard ratio, *CI* confidence interval, *LV* left ventricular, *M* mass (g), *i* indexed to body surface area (m^2^), *EDV* end-diastolic volume (mL), *ESV* end-systolic volume (mL), *EDD* end-diastolic diameter (mm), *EDWT* end-diastolic wall thickness (mm), *EF* ejection fraction (%), *SD* standard deviation

a Group defined by the standard deviation threshold: ≤1 SD (participants with values ≤ mean + 1*SD), >1 SD, ≤2 SD (participants with values > mean + 1*SD and ≤ mean + 2*SD), >2 SD (participants with a value > mean + 2*SD = upper limit of the normal reference range), ≥−1SD (participants with values ≥ mean - 1*SD, <−1SD, ≥−2SD (participants with values < mean - 1*SD and ≥ mean - 2*SD), <−2SD (participants with values < mean - 2*SD = lower limit of the normal reference range).

b n = absolute number of events that occurred in the group, % = participants with events of all participants in the group

#### 3.4.1. LV volume parameters

BSA-adjusted LV end-diastolic and end-systolic volumes were associated with MACE and ACE for values above the upper limit of the normal reference range (> mean + 2*SD) in men and women at 5 and 10 years. Compared to female participants with a LVEDVi below the 1SD-threshold (≤ mean + 1*SD), the risk of MACE was 4.1-fold greater after 5 years of follow-up for women with a LVEDVi above the 2 SD-threshold (HR 4.1, 95% CI 2.1–8.3; *P*<.001). For male participants, the risk for LVEDVi was 4.3-fold higher (HR 4.3, 95% CI 2.3–8.1; *P*<.001). At 5 and 10-year follow-up, there was no association of LVEDVi and LVESVi with MACE and ACE for values at the 1SD-cut-off (> mean + 1*SD, ≤ mean 2*SD).

#### 3.4.2. LV diameter

LVEDD was not associated with MACE or ACE in men and women at 5- and 10-year follow-up for values above the 1SD- or the 2SD-threshold (*P*>.05 for all analyses) (Table 4**,** Supplemental Table 4**,** Fig. 2, Fig. 3). Additional calculations for indexed LVEDD did not change the result.

#### 3.4.3. Left ventricular mass

The cumulative event rate for MACE and ACE was greater in women with a LVMi above the 2SD-cut-off compared to women with values below the 1SD-threshold (≤ mean + 1*SD) (Table 4**,** Supplemental Table 4, Fig. 2, Fig. 3). After 5 years of follow-up the risk of MACE was 4.1-fold greater and the risk of ACE was 2.7-fold greater for female participants with a LVMi above the 2 SD-threshold compared to women with a value ≤ mean + 1*SD (HR 4.1, 95% CI 2.4–6.8; *P*<.001 and HR 2.7, 95% CI 1.4–5.2; *P*=.002, respectively). In women, the risk to develop ACE was significantly greater at the 1SD- and at the 2SD-threshold at 10 years of follow-up compared to female participants with a LVMi ≤ mean + 1*SD (HR 1.4, 95% CI 1.0–2.0; *P=*.027 and HR 2.5, 95% CI 1.8–3.5; *P*<.001, respectively). At 5 years of follow-up, the risk of MACE and ACE was not significantly elevated for women with a LVMi below the 2SD-upper limit. In men, however, the risk of MACE and ACE was significantly greater for participants with a LVMi at the 1SD- and at the 2SD-cut-off at 5 and 10 years of follow-up (*P* between .007 and <.001). Male participants with a LVMi above the 2SD-threshold had a 5.6-fold greater risk to develop MACE compared to men with a value ≤ mean + 1*SD (HR 5.6, 95% CI 3.6–8.8; *P*<.001).

#### 3.4.4. LVED wall thickness

LVED wall thickness was associated with MACE and ACE for values above the 1- SD and 2-SD thresholds in men and women at 5 and 10-year follow-up (*P* between .034 and <.001; Table 4 **and** Supplemental Table 4).

#### 3.4.5. LV ejection fraction

Cumulative event rates for MACE and ACE were higher for men and women with a LVEF below the lower limit (2SD-threshold) but not for values above the 2SD-threshold (Table 4**,** Supplemental Table 4, Fig. 2, Fig. 3). After 5 years of follow-up, the risk to develop MACE was 3.6-fold higher in men and women with a LVEF below the 2SD-threshold compared to participants with values below the 1SD-threshold (< mean - 1*SD, ≥ mean - 2*SD) (HR 3.6, 95% CI 2.1–6.2; *P*<.001 and HR 3.6, 95% CI 1.7–7.5; *P=*.001, respectively).

#### 3.4.6. RV parameters

The number of participants beyond the 1- and 2-standard deviation-thresholds are shown in Table 3 and absolute values of SD-thresholds for RV parameters are shown in Supplemental Table 3. RVEDVi greater than 1 SD was associated with ACE in men, but not at 2 SD, suggesting a weak or spurious association. Furthermore, none of the RV parameters showed significant relationships to ACE or MACE at the 1SD- or 2SD-threshold over 5 or 10 years of follow-up. Full results are shown in Supplemental Tables 5 and 6**.**

### 3.5. Sensitivity analysis

Sensitivity assessment performed by excluding the subpopulation used for normal value determination (derivation set), with remaining MESA participation used as a validation set, yielded similar results.

Additional assessment of risk prediction by comparing the risk for CV events of participants with a LVMi, LVEDVi, LVESVi, LVEDD, and LVED wall thickness, respectively, above the 2SD-threshold with the risk of participants with values equal to or below the 2SD-threshold (for LVEF <−2SD-threshold versus ≥ −2SD-threshold), revealed similar results (Supplemental Tables 7 and 8).

Indexing to height (in meters) instead of BSA resulted in only two significant changes at 10-year follow-up: In female participants, LVEDVi was no longer associated with ACE at a cut-off of >2 SD (HR 1.6, 95% CI 1.0–2.6; *P*=0.064). In male participants, LVESVi was no longer associated with MACE at a cut-off of >1 SD (HR 1.0, 95% CI 0.7–1.6; *P*=0.849).

*Adding additional covariates (age and ethnicity) to the Cox models resulted in minor changes only for females, in all cases reflecting intermediate levels of ventricular remodeling/ function at the 1 SD to 2 SD cut-offs.*

## 4. Discussion

In population-based cardiovascular research, the research intention is typically to identify novel risk factors for CV events above and beyond known/ traditional risk factors. In such studies, the use of threshold values is undesirable (due to lower statistical power) and adjustment for covariates is essential to provide evidence that the new risk factor has an association “independent” of conventional risk factors [8], [10], [11], [12]. However, in the current study, we had a different intention and approach. In clinical CMR-practice, parameters representing cardiac size and systolic function are routinely compared to normal reference ranges established on healthy reference cohorts by the “2SD-method". The clinical significance of values beyond the upper and lower limits, respectively, of these reference ranges has not previously been assessed in terms of risk for future CV events. Further, optimal normal value cut-offs besides the ”2SD-method” have not been evaluated.

In this study, we used the well-characterized MESA cohort to test these assumptions. We derived “normal” mean LV parameters for men and women without conventional CV risk factors. For LV volumes (LVEDVi, LVESVi) and global function (LVEF), CV risk for events was statistically significant beyond the 2-SD-cut-off (lower and upper limit, respectively) in men and women at 5- and 10-year follow-up but not at the 1SD-cut-off (mean ± 1*SD). Parameters related to LV-hypertrophy (LVMi and LVED wall thickness) were associated with CV events at a threshold of 1 SD in men. In women, this was also the case for LVED wall thickness, while LVMi was associated with events only for values above the 2 SD upper limit of the normal reference range. Interestingly, LV diameter was not associated with events at a cut-off of 1 SD and 2 SD in men or women. This result for the left ventricle generally supports the current practice of normal values based on the “2 SD method.”

Our results also confirm intuition regarding directionality of the cut-off values. For example, LVEDVi and LVESVi are associated with an increased risk for MACE and ACE for values above the upper limit of the healthy reference cohort, but not for values below the upper limit. LVEF, an imaging marker of global systolic LV function, has been shown to predict CV events and served as imaging biomarker in several heart failure trials [13], [14]. In addition, we demonstrated in the current study that the risk for CV events was elevated in participants with an LVEF below the 2 SD lower limit of the reference cohort.

Our data provides justification for alternative cut-off values for some LV parameters. LV mass is known to be a strong risk factor for CV events [8], [12], [15], [16]. For men, statistically elevated risk begins at only 1 SD above the mean value, rather than at 2SD-threshold observed for women. In the TASCFORCE study, Weir-McCall et al. showed an independent association of LVM with CV events in men, but not in women [17]. Similarly, LVED wall thickness, another marker of LV hypertrophy, was associated with CV events in men and women for values beginning at a threshold of 1 SD above mean normal values. Lundin et al. recently also identified LVED wall thickness as valuable predictor of CV events in 1575 patients over a median follow-up of 5.4 years [16].

Mainly in echocardiography-based studies, LVEDD was identified as predictor of CV events [18], [19]. However, in the current study, CMR-derived LVEDD was neither in male nor in female participants associated with events for values above and below the 2 SD upper limit. The routine inclusion of cut-off values for LV end-diastolic diameter is not supported by our data.

In previous analyses of the MESA cohort, allometric indexing of RV mass and RVESV above the 95th percentile have been shown to predict selected events (heart failure and CV death) [20]. In the current study, however, RV parameters of volume and function indexed by BSA were not related to the same, broader definition of cardiovascular events also used for the left ventricle. RV parameters may be more sensitive to methods of body size correction and/ or more strongly related to specific event types. The thinner wall and irregular contour of the right ventricle compared to the left ventricle can cause greater measurement variability and thus impair the analysis. Further, it might be possible that RV parameters become prognostic significant at higher thresholds (e.g., mean + 3*SD), which could not be assessed in the current study due to the insufficient number of participants and events at higher thresholds.

In routine clinical application, the interpretation of CMR reference values in adults may eventually evolve to consider other factors, such as age or ethnicity. Nevertheless, the results of this study support the use of CMR cut-off values as applied in current clinical practice.

## 5. Limitations

This study has several limitations. There is no general agreement as to the level of elevated risk (e.g., 2-fold, 4-fold, etc) that is clinically significant (as opposed to statistical significance). Furthermore, the change in volume or mass values over time may be as or more important than values at one point in time. These limitations are, however, common to nearly all similar studies seeking to identify normal values of a population.

Absolute values for CMR parameters depend on multiple factors, including the CMR pulse sequence, the validity of assumed slice-to-slice independence, the CMR analyst, and the CMR analysis software. To mitigate these effects, we analyzed the relationship of groups defined by standard deviation thresholds instead of absolute values with events, for which the underlying CMR technology is likely irrelevant. According to clinical practice, we used BSA to adjust for body size. Research studies on CVMR risk factors have used allometric methods to adjust for body size [5]. Allometric methods have the disadvantage of requiring a unique index for each individual RV and LV parameter and are thus cumbersome for clinical implementation. Finally, we note that the relationship of CMR parameters to CV events was assessed without adjustment for CV risk factors (e.g., diabetes, hypertension). This is in keeping with clinical practice, where patients’ values are compared to reference ranges independent of known CV risk factors. Adjustment for risk factors was thus not relevant to the study aim. Given their greater relevance in routine clinical practice, only parameters of right and left ventricular volume and function were subject of the current study. However, parameters of atrial size and function should also be examined analogously. We also point out that the results of the current study require validation in additional cohorts.

## 6. Conclusion

This study confirms the clinical approach of calculating left ventricular reference ranges for CMR calculated as mean values plus or minus 2 standard deviations. Beyond those limits, an elevated risk for cardiovascular events was evident, suggesting that MR may be identifying occult cardiovascular disease in subjects with no known cardiac disease and free of risk factors. For LV mass and wall thickness, elevated cardiovascular risk was present when values exceeded 1 standard deviation of the mean value. Particular attention to these parameters using lower than commonly employed clinical cut-offs may be warranted. To increase the clinical relevance of reference intervals of quantitative parameters, in future research, upper and lower limits, respectively, could be defined not only based on the values of healthy reference cohorts but also by considering association with events.

## Funding

This research was supported by contracts 75N92020D00001, HHSN268201500003I, N01-HC-95159, 75N92020D00005, N01-HC-95160, 75N92020D00002, N01-HC-95161, 75N92020D00003, N01-HC-95162, 75N92020D00006, N01-HC-95163, 75N92020D00004, N01-HC-95164, 75N92020D00007, N01-HC-95165, N01-HC-95166, N01-HC-95167, N01-HC-95168 and N01-HC-95169 from the 10.13039/100000050National Heart, Lung, and Blood Institute, and by grants UL1-TR-000040, UL1-TR-001079, and UL1-TR-001420 from the National Center for Advancing Translational Sciences (NCATS). A full list of participating MESA investigators and institutions can be found at http://www.mesa-nhlbi.org.

## Author contributions

**Nadine Kawel-Boehm:** Writing – review & editing, writing – original draft, methodology, investigation, conceptualization. **Spencer L. Hansen:** Writing – review & editing, methodology, investigation, formal analysis, conceptualization. **Bharath Ambale-Venkatesh:** Writing – review & editing, investigation. **J. Jeffrey Carr:** Writing – review & editing, investigation. **J. Paul Finn:** Writing – review & editing, investigation. **Michael Jerosch-Herold:** Writing – review & editing, investigation. **Steven M. Kawut:** Writing – review & editing, investigation. **Robyn L. McClelland:** Writing – review & editing, methodology, investigation, formal analysis. **Wendy Post:** Writing – review & editing, investigation. **Martin R. Prince:** Writing – review & editing, investigation. **Steven Shea:** Writing – review & editing, investigation. **João A.C. Lima:** Writing – review & editing, investigation. **David A. Bluemke:** Writing – review & editing, writing – original draft, methodology, investigation, conceptualization.

## Additional contributions

We thank the investigators, staff, and participants of the MESA study for their contributions.

## Declaration of competing interests

The authors declare the following financial interests/personal relationships which may be considered as potential competing interests: David Bluemke is a consultant for GE HealthCare. Martin Prince has a patent agreement with GE HealthCare. The remaining authors declare that they have no known competing financial interests or personal relationships that could have appeared to influence the work reported in this paper.

## Declaration of Generative AI and AI-assisted technologies in the writing process

The authors confirm that they made no use of generative AI and AI-assisted technologies in the writing process.

## Data Availability

MESA data are publicly available to investigators via BioLINCC or through the MESA Coordinating Center subject to MESA policies and participant consents.
