# Supplementary material for: Validation of normal reference ranges in cardiac magnetic resonance imaging: The Multi-Ethnic Study of Atherosclerosis
Source: J Cardiovasc Magn Reson. 2025 Aug 26;27(2):101949. doi: 10.1016/j.jocmr.2025.101949 (PMC12670894; doi:10.1016/j.jocmr.2025.101949)
Supplement: Supplementary file 1 — Supplementary material [file mmc1.docx]

**Supplemental tables**

**Supplemental Table 1** Baseline characteristics of the MESA study cohort for analysis of RV parameters

| **Characteristic** | **Entire cohort**  **(n=4146)** | **Participants without CV risk factors (n=1257)** | **Participants with CV risk factors (n=2889)** |
| --- | --- | --- | --- |
| Age, mean (SD), years | 61.4 (10.1) | 58.8 (10.0) | 62.6 (9.9) |
| Sex, No. (%) |  |  |  |
| Women | 2178 (53%) | 752 (60%) | 1426 (49%) |
| Men | 1968 (47%) | 505 (40%) | 1463 (51%) |
| Race/ ethnicity, No. (%) |  |  |  |
| White | 1624 (39%) | 568 (46%) | 1056 (57%) |
| Chinese | 520 (13%) | 213 (17%) | 307 (11%) |
| Black/ African American | 1085 (26%) | 236 (18%) | 849 (29%) |
| Hispanic | 917 (22%) | 240 (19%) | 677 (23%) |
| Height, mean (SD), m | 1.7 (0.1) | 1.7 (0.1) | 1.7 (0.1) |
| Weight, mean (SD), kg | 77.4 (16.2) | 73.1 (15.7) | 79.2 (16.0) |
| Body mass index, mean (SD), kg/m^2^ | 27.9 (5.0) | 26.4 (4.7) | 28.5 (5.0) |
| Body surface area, mean (SD), m^2^ | 1.9 (0.2) | 1.8 (0.2) | 1.9 (0.2) |
| Cholesterol, mean (SD), mg/dL |  |  |  |
| Total | 194 (35) | 191 (26) | 196 (38) |
| HDL | 51 (15) | 57 (14) | 49 (15) |
| Fasting glucose, mean (SD), mg/dl | 96 (29) | 86 (9) | 101 (33) |
| Diabetic medication use, No. (%) |  |  |  |
| No | 3770 (91%) | 1257 (100%) | 2513 (87%) |
| Yes | 376 (9%) | 0 (0%) | 376 (13%) |
| Blood pressure, mean (SD), mmHg |  |  |  |
| Systolic | 125 (21) | 114 (13) | 130 (22) |
| Diastolic | 72 (10) | 69 (9) | 73 (10) |
| Hypertension medication use, No. (%) |  |  |  |
| No | 2658 (64%) | 1257 (100%) | 1401 (48%) |
| Yes | 1488 (36%) | 0 (0%) | 1488 (52%) |
| Smoking status, No. (%) |  |  |  |
| Never | 2142 (52%) | 770 (61%) | 1372 (47%) |
| Former smoker | 1483 (36%) | 487 (39%) | 996 (34%) |
| Current smoker | 521 (13%) | 0 (0%) | 521 (18%) |

CV = cardiovascular; SD = 1 standard deviation; HDL = high density lipoprotein

**Supplemental Table 2** Number of individual cardiovascular events

|  | **Female** (n = 2575) | | **Male** (n = 2337) | |
| --- | --- | --- | --- | --- |
| **Follow-up** | **5-year** | **10-year** | **5-year** | **10-year** |
| Myocardial infarction | 12 | 30 | 43 | 67 |
| Resuscitated cardiac arrest | 0 | 0 | 1 | 3 |
| CVD related death | 6 | 19 | 10 | 33 |
| Stroke | 29 | 54 | 20 | 51 |
| Heart failure | 22 | 38 | 36 | 61 |
| Combination | 11 | 20 | 11 | 35 |
| **MACE** | **80** | **161** | **122** | **250** |
|  |  |  |  |  |
| Angina | 27 | 36 | 52 | 60 |
| Peripheral vascular disease | 3 | 9 | 16 | 20 |
| PTCA | 2 | 3 | 6 | 16 |
| CABG | 0 | 2 | 4 | 7 |
| Other revascularization | 1 | 1 | 3 | 2* |
| Transient ischemic attack | 4 | 20 | 10 | 17 |
| Combination | 10 | 15 | 13 | 24 |
| **ACE**** | **127** | **247** | **226** | **396** |

CVD = cardiovascular disease; MACE = major adverse cardiovascular events; PTCA = percutaneous transluminal coronary angioplasty; CABG = coronary artery bypass grafting;

ACE = all cardiovascular events

*An individual that had “other revascularization” at 5-years had a MACE event at 10-years which explains the decrease of 1; ** includes MACE

**Supplemental Table 3** Standard deviation-thresholds for CMR-derived ^1^ variables of left and right ventricular size and function

|  | **Female** (n = 919) ^2^ | | | **Male** (n = 609) ^2^ | | |
| --- | --- | --- | --- | --- | --- | --- |
| **Variable** | **mean** | **+ 1SD** | **+ 2SD** | **mean** | **+ 1SD** | **+ 2SD** |
| LVMi (g/m^2^) | 67 | 77 | 87 | 83 | 97 | 111 |
| LVEDVi (ml/m^2^) | 66 | 77 | 87 | 74 | 88 | 102 |
| LVESVi (ml/m^2^) | 19 | 25 | 31 | 25 | 33 | 41 |
| LVEDD (mm/m^2^) | 44 | 49 | 55 | 47 | 53 | 59 |
| LVED wall thickness (mm) | 8.0 | 9.3 | 10.6 | 9.6 | 11.2 | 12.9 |
| RVMi (g/m^2^) | 11 | 13 | 15 | 12 | 14 | 16 |
| RVEDVi (ml/m^2^) | 63 | 74 | 84 | 74 | 87 | 100 |
| RVESVi (ml/m^2^) | 18 | 23 | 28 | 24 | 31 | 38 |
|  |  |  |  |  |  |  |
| **Variable** | **mean** | **-1SD** | **-2SD** | **mean** | **-1SD** | **-2SD** |
| LVEF (%) | 71 | 65 | 59 | 67 | 60 | 53 |
| RVEF (%) | 72 | 67 | 61 | 68 | 62 | 55 |

LV = left ventricular; M = mass; i = indexed to body surface area; EDV = end-diastolic volume; ESV = end-systolic volume; EDD = end-diastolic diameter; RV = right ventricular; EF = ejection fraction

^1^ data derived from gradient echo cine imaging

^2^ without known cardiovascular disease and risk factors

**Supplemental Table 4** Relationship of left ventricular CMR variables to cardiovascular events at 10-year follow-up assessed by the Cox proportional hazards model (unadjusted)

|  | **Female** (n = 2575) | | | | | | **Male** (n = 2337) | | | | | |
| --- | --- | --- | --- | --- | --- | --- | --- | --- | --- | --- | --- | --- |
|  | **MACE** | | | **ACE** | | | **MACE** | | | **ACE** | | |
| **Variable**  Group ^1^ | **Events**  **n (%)** ^2^ | **HR (95% CI)** | ***P*** | **Events**  **n (%)** ^2^ | **HR (95% CI)** | ***P*** | **Events**  **n (%)** ^2^ | **HR (95% CI)** | ***P*** | **Events**  **n (%)** ^2^ | **HR (95% CI)** | ***P*** |
| **LVMi** |  |  |  |  |  |  |  |  |  |  |  |  |
| ≤1SD | 96 (5) | Reference |  | 151 (8) | Reference |  | 157 (8) | Reference |  | 267 (14) | Reference |  |
| >1SD, ≤2SD | 31 (7) | 1.3 (0.9-2.0) | 0.154 | 52 (11) | 1.4 (1.0-2.0) | **0.027** | 43 (13) | 1.6 (1.1-2.2) | **0.007** | 69 (21) | 1.5 (1.2-2.0) | **0.002** |
| >2SD | 34 (14) | 3.0 (2.0-4.4) | **<0.001** | 44 (18) | 2.5 (1.8-3.5) | **<0.001** | 50 (34) | 4.8 (3.5-6.6) | **<0.001** | 60 (41) | 3.5 (2.7-4.6) | **<0.001** |
| **LVEDVi** |  |  |  |  |  |  |  |  |  |  |  |  |
| ≤1SD | 135 (6) | Reference |  | 214 (10) | Reference |  | 212 (10) | Reference |  | 342 (17) | Reference |  |
| >1SD, ≤2SD | 14 (5) | 0.8 (0.4-1.4) | 0.453 | 19 (7) | 0.7 (0.4-1.1) | 0.107 | 19 (9) | 0.9 (0.6-1.4) | 0.604 | 32 (15) | 0.9 (0.6-1.3) | 0.689 |
| >2SD | 12 (15) | 2.6 (1.4-4.7) | **0.001** | 14 (17) | 1.9 (1.1-3.3) | **0.016** | 19 (32) | 3.8 (2.3-6.0) | **<0.001** | 22 (37) | 2.7 (1.8-4.2) | **<0.001** |
| **LVESVi** |  |  |  |  |  |  |  |  |  |  |  |  |
| ≤1SD | 137 (6) | Reference |  | 211 (10) | Reference |  | 196 (10) | Reference |  | 323 (16) | Reference |  |
| >1SD, ≤2SD | 13 (5) | 0.8 (0.5-1.4) | 0.434 | 23 (9) | 0.9 (0.6-1.4) | 0.709 | 28 (14) | 1.5 (1.0-2.3) | **0.038** | 37 (19) | 1.2 (0.9-1.7) | 0.276 |
| >2SD | 11 (12) | 2.1 (1.1-3.8) | **0.020** | 13 (14) | 1.6 (0.9-2.8) | 0.114 | 26 (30) | 3.5 (2.3-5.5) | **<0.001** | 36 (41) | 3.3 (2.4-4.7) | **<0.001** |
| **LVEDD** |  |  |  |  |  |  |  |  |  |  |  |  |
| ≤1SD | 141 (6) | Reference |  | 212 (9) | Reference |  | 217 (11) | Reference |  | 347 (17) | Reference |  |
| >1SD, ≤2SD | 16 (6) | 0.9 (0.5-1.5) | 0.698 | 30 (11) | 1.1 (0.8-1.5) | 0.553 | 26 (10) | 1.0 (0.6-1.5) | 0.879 | 39 (15) | 0.9 (0.6-1.3) | 0.545 |
| >2SD | 4 (6) | 1.0 (0.4-2.7) | 0.973 | 5 (8) | 0.8 (0.3-2.2) | 0.639 | 7 (17) | 1.6 (0.8-3.4) | 0.220 | 10 (24) | 1.4 (0.7-2.6) | 0.306 |
| **LVEDWT** |  |  |  |  |  |  |  |  |  |  |  |  |
| ≤1SD | 89 (5) | Reference |  | 140 (8) | Reference |  | 154 (9) | Reference |  | 262 (15) | Reference |  |
| >1SD, ≤2SD | 41 (9) | 1.9 (1.3-2.7) | **0.001** | 65 (14) | 1.9 (1.4-2.5) | **<0.001** | 58 (15) | 1.8 (1.3-2.4) | **<0.001** | 86 (22) | 1.6 (1.3-2.0) | **<0.001** |
| >2SD | 31 (13) | 3.0 (2.0-4.6) | **<0.001** | 42 (18) | 2.7 (1.9-3.8) | **<0.001** | 38 (22) | 2.8 (2.0-4.0) | **<0.001** | 48 (27) | 2.1 (1.5-2.8) | **<0.001** |
| **LVEF** |  |  |  |  |  |  |  |  |  |  |  |  |
| ≥-1SD | 126 (6) | Reference |  | 200 (9) | Reference |  | 190 (10) | Reference |  | 313 (16) | Reference |  |
| <-1SD, ≥-2SD | 24 (8) | 1.5 (1.0-2.3) | 0.073 | 33 (12) | 1.3 (0.9-1.9) | 0.159 | 33 (12) | 1.3 (0.9-1.8) | 0.237 | 45 (16) | 1.0 (0.8-1.4) | 0.831 |
| <-2SD | 11 (13) | 2.5 (1.4-4.6) | **0.004** | 14 (17) | 2.0 (1.2-3.5) | **0.012** | 27 (29) | 3.5 (2.3-5.3) | **<0.001** | 38 (40) | 3.4 (2.4-4.7) | **<0.001** |

MACE = major adverse cardiovascular events: myocardial infarction (MI), resuscitated cardiac arrest, stroke, mortality related to coronary heart disease (CHD), cardiovascular disease (CVD), arteriosclerotic disease or stroke, heart failure; ACE = all cardiovascular events: MACE, angina, peripheral vascular disease (PVD), percutaneous transluminal coronary angioplasty (PTCA), coronary artery bypass grafting (CABG), transient ischemic attack (TIA); HR = hazard ratio; CI = confidence interval; LV = left ventricular; M = mass (g); i = indexed to body surface area (m^2^); EDV = end-diastolic volume (ml); ESV = end-systolic volume (ml); EDD = end-diastolic diameter (mm); EDWT = end-diastolic wall thickness (mm); EF = ejection fraction (%)

^1^ Group defined by the standard deviation threshold: ≤1SD (participants with values ≤ mean + 1*SD), >1SD, ≤2SD (participants with values > mean + 1*SD and ≤ mean + 2*SD), >2SD (participants with a value > mean + 2*SD = upper limit of the normal reference range), ≥-1SD (participants with values ≥ mean - 1*SD, <-1SD, ≥-2SD (participants with values < mean - 1*SD and ≥ mean - 2*SD), <-2SD (participants with values < mean - 2*SD = lower limit of the normal reference range).

^2^ n = absolute number of events that occurred in the group, % = participants with events of all participants in the group

**Supplemental Table 5** Relationship of right ventricular CMR variables to cardiovascular events at 5-year follow-up assessed by the Cox proportional hazards model (unadjusted).

|  | **Female** (n = 2178) | | | | | | **Male** (n = 1968) | | | | | |
| --- | --- | --- | --- | --- | --- | --- | --- | --- | --- | --- | --- | --- |
|  | **MACE** | | | **ACE** | | | **MACE** | | | **ACE** | | |
| **Variable**  Group ^1^ | **Events**  **n (%)** ^2^ | **HR (95% CI)** | ***P*** | **Events**  **n (%)** ^2^ | **HR (95% CI)** | ***P*** | **Events**  **n (%)** ^2^ | **HR (95% CI)** | ***P*** | **Events**  **n (%)** ^2^ | **HR (95% CI)** | ***P*** |
| **RVMi** |  |  |  |  |  |  |  |  |  |  |  |  |
| ≤1SD | 64 (4) | Reference |  | 105 (6) | Reference |  | 88 (5) | Reference |  | 176 (10) | Reference |  |
| >1SD, ≤2SD | 7 (3) | 0.9 (0.4-1.9) | 0.752 | 10 (2) | 0.8 (0.4-1.5) | 0.423 | 10 (6) | 1.1 (0.6-2.2) | 0.729 | 12 (7) | 0.7 (0.4-1.2) | 0.167 |
| >2SD | 1 (9) | 0.5 (2.4-6.8) | 0.504 | 1 (2) | 0.3 (0.0-2.2) | 0.247 | 3 (7) | 1.4 (0.4-4.4) | 0.578 | 5 (12) | 1.2 (0.5-2.9) | 0.712 |
| **RVEDVi** |  |  |  |  |  |  |  |  |  |  |  |  |
| ≤1SD | 61 (3) | Reference |  | 103 (5) | Reference |  | 89 (5) | Reference |  | 179 (10) | Reference |  |
| >1SD, ≤2SD | 11 (4) | 1.4 (0.5-2.1) | 0.356 | 13 (5) | 0.9 (0.5-1.7) | 0.840 | 8 (4) | 0.9 (0.4-1.8) | 0.722 | 9 (5) | 0.5 (0.2-0.9) | **0.032** |
| >2SD | 0 (0) | - | - | 0 (0) | - | - | 4 (11) | 2.1 (0.8-5.8) | 0.139 | 5 (13) | 1.3 (0.5-3.2) | 0.525 |
| **RVESVi** |  |  |  |  |  |  |  |  |  |  |  |  |
| ≤1SD | 66 (4) | Reference |  | 109 (6) | Reference |  | 87 (5) | Reference |  | 172 (10) | Reference |  |
| >1SD, ≤2SD | 4 (2) | 0.5 (0.2-1.4) | 0.176 | 7 (3) | 0.5 (0.3-1.2) | 0.111 | 12 (7) | 1.3 (0.7-2.4) | 0.360 | 18 (10) | 1.0 (0.6-1.6) | 0.945 |
| >2SD | 2 (3) | 0.7 (0.2-3.0) | 0.651 | 2 (3) | 0.4 (0.1-1.8) | 0.245 | 2 (4) | 0.9 (0.2-3.5) | <0.829 | 3 (6) | 0.7 (0.2-2.0) | 0.464 |
| **RVEF** |  |  |  |  |  |  |  |  |  |  |  |  |
| ≥-1SD | 61 (3) | Reference |  | 101 (5) | Reference |  | 83 (5) | Reference |  | 171 (10) | Reference |  |
| <-1SD, ≥-2SD | 8 (3) | 1.0 (0.5-2.1) | 0.971 | 11 (5) | 0.8 (0.5-1.6) | 0.583 | 14 (6) | 1.3 (0.7-2.2) | 0.426 | 27 (12) | 1.3 (0.8-1.9) | 0.266 |
| <-2SD | 3 (4) | 1.1 (0.3-3.5) | 0.876 | 4 (5) | 0.9 (0.3-2.4) | 0.804 | 4 (8) | 1.6 (0.6-4.2) | 0.389 | 5 (9) | 1.0 (0.4-2.4) | 0.985 |

MACE = major adverse cardiovascular events: myocardial infarction (MI), resuscitated cardiac arrest, stroke, mortality related to coronary heart disease (CHD), cardiovascular disease (CVD), arteriosclerotic disease or stroke, heart failure; ACE = all cardiovascular events: MACE, angina, peripheral vascular disease (PVD), percutaneous transluminal coronary angioplasty (PTCA), coronary artery bypass grafting (CABG), transient ischemic attack (TIA); HR = hazard ratio; CI = confidence interval; RV = right ventricular; M = mass (g); i = indexed to body surface area (m^2^); EDV = end-diastolic volume (ml); ESV = end-systolic volume (ml); EF = ejection fraction (%)

^1^ Group defined by the standard deviation threshold: ≤1SD (participants with values ≤ mean + 1*SD), >1SD, ≤2SD (participants with values > mean + 1*SD and ≤ mean + 2*SD), >2SD (participants with a value > mean + 2*SD = upper limit of the normal reference range), ≥-1SD (participants with values ≥ mean - 1*SD, <-1SD, ≥-2SD (participants with values < mean - 1*SD and ≥ mean - 2*SD), <-2SD (participants with values < mean - 2*SD = lower limit of the normal reference range).

^2^ n = absolute number of events that occurred in the group, % = participants with events of all participants in the group

**Supplemental Table 6** Relationship of right ventricular CMR variables to cardiovascular events at 10-year follow-up assessed by the Cox proportional hazards model (unadjusted)

|  | **Female** (n = 2178) | | | | | | **Male** (n = 1968) | | | | | |
| --- | --- | --- | --- | --- | --- | --- | --- | --- | --- | --- | --- | --- |
|  | **MACE** | | | **ACE** | | | **MACE** | | | **ACE** | | |
| **Variable**  Group ^1^ | **Events**  **n (%)** ^2^ | **HR (95% CI)** | ***P*** | **Events**  **n (%)** ^2^ | **HR (95% CI)** | ***P*** | **Events**  **n (%)** ^2^ | **HR (95% CI)** | ***P*** | **Events**  **n (%)** ^2^ | **HR (95% CI)** | ***P*** |
| **RVMi** |  |  |  |  |  |  |  |  |  |  |  |  |
| ≤1SD | 130 (8) | Reference |  | 201 (12) | Reference |  | 177 (10) | Reference |  | 299 (17) | Reference |  |
| >1SD, ≤2SD | 14 (3) | 0.9 (0.5-1.5) | 0.586 | 20 (5) | 0.8 (0.5-1.3) | 0.328 | 17 (9) | 0.9 (0.6-1.5) | 0.802 | 21 (12) | 0.7 (0.4-1.0) | 0.076 |
| >2SD | 2 (4) | 0.5 (0.1-2.0) | 0.329 | 2 (4) | 0.3 (0.1-1.3) | 0.111 | 3 (7) | 0.7 (0.2-2.1) | 0.510 | 6 (14) | 0.8 (0.4-1.8) | 0.625 |
| **RVEDVi** |  |  |  |  |  |  |  |  |  |  |  |  |
| ≤1SD | 130 (7) | Reference |  | 203 (11) | Reference |  | 176 (10) | Reference |  | 300 (17) | Reference |  |
| >1SD, ≤2SD | 15 (6) | 0.9 (0.5-1.5) | 0.564 | 19 (8) | 0.7 (0.4-1.1) | 0.107 | 15 (8) | 0.8 (0.5-1.4) | 0.442 | 18 (10) | 0.6 (0.3-0.9) | **0.016** |
| >2SD | 1 (2) | 0.3 (0.0-2.0) | 0.212 | 1 (2) | 0.2 (0.0-1.3) | 0.086 | 6 (16) | 1.6 (0.7-3.6) | 0.260 | 8 (21) | 1.2 (0.6-2.5) | 0.536 |
| **RVESVi** |  |  |  |  |  |  |  |  |  |  |  |  |
| ≤1SD | 134 (7) | Reference |  | 202 (11) | Reference |  | 172 (10) | Reference |  | 188 (11) | Reference |  |
| >1SD, ≤2SD | 9 (4) | 0.5 (0.3-1.1) | 0.080 | 15 (7) | 0.6 (0.4-1.0) | 0.059 | 20 (11) | 1.1 (0.7-1.8) | 0.677 | 32 (18) | 1.0 (0.7-1.5) | 0.859 |
| >2SD | 3 (4) | 0.5 (0.2-1.7) | 0.271 | 6 (8) | 0.7 (0.3-1.6) | 0.376 | 5 (11) | 1.1 (0.4-2.6) | 0.882 | 6 (13) | 0.8 (0.3-1.7) | 0.526 |
| **RVEF** |  |  |  |  |  |  |  |  |  |  |  |  |
| ≥-1SD | 121 (7) | Reference |  | 184 (10) | Reference |  | 165 (10) | Reference |  | 281 (17) | Reference |  |
| <-1SD, ≥-2SD | 19 (8) | 1.2 (0.8-2.0) | 0.414 | 29 (12) | 1.2 (0.8-1.8) | 0.305 | 23 (10) | 1.0 (0.7-1.6) | 0.851 | 43 (19) | 1.2 (0.9-1.6) | 0.283 |
| <-2SD | 6 (7) | 1.1 (0.5-2.5) | 0.815 | 10 (12) | 1.2 (0.6-2.3) | 0.538 | 9 (17) | 1.8 (0.9-3.5) | 0.091 | 12 (23) | 1.4 (0.8-2.5) | 0.232 |

MACE = major adverse cardiovascular events: myocardial infarction (MI), resuscitated cardiac arrest, stroke, mortality related to coronary heart disease (CHD), cardiovascular disease (CVD), arteriosclerotic disease or stroke, heart failure; ACE = all cardiovascular events: MACE, angina, peripheral vascular disease (PVD), percutaneous transluminal coronary angioplasty (PTCA), coronary artery bypass grafting (CABG), transient ischemic attack (TIA); HR = hazard ratio; CI = confidence interval; RV = right ventricular; M = mass (g); i = indexed to body surface area (m^2^); EDV = end-diastolic volume (ml); ESV = end-systolic volume (ml); SV = stroke volume (ml); EF = ejection fraction (%)

^1^ Group defined by the standard deviation threshold: ≤1SD (participants with values ≤ mean + 1*SD), >1SD, ≤2SD (participants with values > mean + 1*SD and ≤ mean + 2*SD), >2SD (participants with a value > mean + 2*SD = upper limit of the normal reference range), ≥-1SD (participants with values ≥ mean - 1*SD, <-1SD, ≥-2SD (participants with values < mean - 1*SD and ≥ mean - 2*SD), <-2SD (participants with values < mean - 2*SD = lower limit of the normal reference range).

^2^ n = absolute number of events that occurred in the group, % = participants with events of all participants in the group

**Supplemental Table 7** Relationship of left ventricular CMR variables to cardiovascular events at 5-year follow-up assessed by the Cox proportional hazards model (unadjusted).

|  | **Female** (n = 2575) | | | | | | **Male** (n = 2337) | | | | | |
| --- | --- | --- | --- | --- | --- | --- | --- | --- | --- | --- | --- | --- |
|  | **MACE** | | | **ACE** | | | **MACE** | | | **ACE** | | |
| **Variable**  Group ^1^ | **Events**  **n (%)** ^2^ | **HR (95% CI)** | ***P*** | **Events**  **n (%)** ^2^ | **HR (95% CI)** | ***P*** | **Events**  **n (%)** ^2^ | **HR (95% CI)** | ***P*** | **Events**  **n (%)** ^2^ | **HR (95% CI)** | ***P*** |
| **LVMi** |  |  |  |  |  |  |  |  |  |  |  |  |
| ≤2SD | 58 (3) | Reference |  | 101 (5) | Reference |  | 94 (5) | Reference |  | 189 (10) | Reference |  |
| >2SD | 22 (9) | 3.7 (2.3-6.1) | **<0.001** | 26 (11) | 2.6 (1.7-3.9) | **<0.001** | 28 (19) | 4.8 (3.2-7.4) | **<0.001** | 37 (25) | 3.3 (2.3-4.6) | **<0.001** |
| **LVEDVi** |  |  |  |  |  |  |  |  |  |  |  |  |
| ≤2SD | 71 (3) | Reference |  | 117 (5) | Reference |  | 111 (5) | Reference |  | 212 (10) | Reference |  |
| >2SD | 9 (11) | 4.1 (2.1-8.3) | **<0.001** | 10 (12) | 2.8 (1.5-5.3) | **0.002** | 11 (19) | 4.2 (2.3-7.9) | **<0.001** | 14 (24) | 2.9 (1.7-5.0) | **<0.001** |
| **LVESVi** |  |  |  |  |  |  |  |  |  |  |  |  |
| ≤2SD | 72 (3) | Reference |  | 118 (5) | Reference |  | 108 (5) | Reference |  | 202 (10) | Reference |  |
| >2SD | 8 (9) | 3.2 (1.5-6.6) | **0.002** | 9 (10) | 2.2 (1.1-4.3) | **0.026** | 14 (16) | 3.6 (2.1-6.4) | **<0.001** | 24 (28) | 3.6 (2.3-5.4) | **<0.001** |
| **LVEDD** |  |  |  |  |  |  |  |  |  |  |  |  |
| ≤2SD | 77 (3) | Reference |  | 123 (6) | Reference |  | 118 (6) | Reference |  | 222 (11) | Reference |  |
| >2SD | 3 (5) | 1.5 (0.5-4.9) | 0.463 | 4 (6) | 1.3 (0.5-3.5) | 0.634 | 4 (10) | 1.9 (0.7-5.2) | 0.204 | 4 (10) | 1.0 (0.4-2.7) | 0.991 |
| **LVEDWT** |  |  |  |  |  |  |  |  |  |  |  |  |
| ≤2SD | 63 (3) | Reference |  | 105 (6) | Reference |  | 97 (5) | Reference |  | 194 (11) | Reference |  |
| >2SD | 17 (7) | 2.8 (1.6-4.7) | **<0.001** | 22 (9) | 2.2 (1.4-3.5) | **0.001** | 25 (14) | 3.4 (2.2-5.2) | **<0.001** | 32 (18) | 2.2 (1.5-3.1) | **<0.001** |
| **LVEF** |  |  |  |  |  |  |  |  |  |  |  |  |
| ≥-2SD | 72 (3) | Reference |  | 118 (5) | Reference |  | 107 (5) | Reference |  | 202 (10) | Reference |  |
| <-2SD | 8 (10) | 3.5 (1.7-7.3) | **0.001** | 9 (11) | 2.4 (1.2-4.7) | **0.011** | 15 (16) | 3.6 (2.1-6.2) | **<0.001** | 24 (26) | 3.3 (2.2-5.0) | **<0.001** |

MACE = major adverse cardiovascular events: myocardial infarction (MI), resuscitated cardiac arrest, stroke, mortality related to coronary heart disease (CHD), cardiovascular disease (CVD), arteriosclerotic disease or stroke, heart failure; ACE = all cardiovascular events: MACE, angina, peripheral vascular disease (PVD), percutaneous transluminal coronary angioplasty (PTCA), coronary artery bypass grafting (CABG), transient ischemic attack (TIA); HR = hazard ratio; CI = confidence interval; LV = left ventricular; M = mass (g); i = indexed to body surface area (m^2^); EDV = end-diastolic volume (ml); ESV = end-systolic volume (ml); EDD = end-diastolic diameter (mm); EDWT = end-diastolic wall thickness (mm); EF = ejection fraction (%)

^1^ Group defined by the standard deviation threshold: ≤2SD (participants with values ≤ mean + 2*SD), >2SD (participants with a value > mean + 2*SD = upper limit of the normal reference range), ≥-2SD (participants with values ≥ mean - 2*SD), <-2SD (participants with values < mean - 2*SD = lower limit of the normal reference range).

^2^ n = absolute number of events that occurred in the group, % = participants with events of all participants in the group

**Supplemental Table 8** Relationship of left ventricular CMR variables to cardiovascular events at 10-year follow-up assessed by the Cox proportional hazards model (unadjusted)

|  | **Female** (n = 2575) | | | | | | **Male** (n = 2337) | | | | | |
| --- | --- | --- | --- | --- | --- | --- | --- | --- | --- | --- | --- | --- |
|  | **MACE** | | | **ACE** | | | **MACE** | | | **ACE** | | |
| **Variable**  Group ^1^ | **Events**  **n (%)** ^2^ | **HR (95% CI)** | ***P*** | **Events**  **n (%)** ^2^ | **HR (95% CI)** | ***P*** | **Events**  **n (%)** ^2^ | **HR (95% CI)** | ***P*** | **Events**  **n (%)** ^2^ | **HR (95% CI)** | ***P*** |
| **LVMi** |  |  |  |  |  |  |  |  |  |  |  |  |
| ≤2SD | 127 (7) | Reference |  | 203 (11) | Reference |  | 200 (11) | Reference |  | 336 (18) | Reference |  |
| >2SD | 34 (14) | 2.8 (1.9-4.1) | **<0.001** | 44 (18) | 2.3 (1.7-3.2) | **<0.001** | 50 (34) | 4.4 (3.2-6.0) | **<0.001** | 60 (41) | 3.3 (2.5-4.3) | **<0.001** |
| **LVEDVi** |  |  |  |  |  |  |  |  |  |  |  |  |
| ≤2SD | 149 (7) | Reference |  | 233 (11) | Reference |  | 231 (11) | Reference |  | 374 (18) | Reference |  |
| >2SD | 12 (15) | 2.7 (1.5-4.8) | **0.001** | 14 (17) | 2.0 (1.2-3.5) | **0.011** | 19 (32) | 3.8 (2.4-6.1) | **<0.001** | 22 (37) | 2.8 (1.8-4.3) | **<0.001** |
| **LVESVi** |  |  |  |  |  |  |  |  |  |  |  |  |
| ≤2SD | 150 (7) | Reference |  | 234 (11) | Reference |  | 224 (11) | Reference |  | 360 (18) | Reference |  |
| >2SD | 11 (12) | 2.1 (1.2-3.9) | **0.016** | 13 (14) | 1.6 (0.9-2.8) | 0.106 | 26 (30) | 3.5 (2.3-5.2) | **<0.001** | 36 (41) | 3.3 (2.3-4.6) | **<0.001** |
| **LVEDD** |  |  |  |  |  |  |  |  |  |  |  |  |
| ≤2SD | 157 (7) | Reference |  | 242 (11) | Reference |  | 243 (12) | Reference |  | 386 (19) | Reference |  |
| >2SD | 4 (6) | 1.0 (0.4-2.7) | 0.991 | 5 (8) | 0.8 (0.3-1.9) | 0.618 | 7 (17) | 1.6 (0.8-3.4) | 0.216 | 10 (24) | 1.4 (0.8-2.6) | 0.290 |
| **LVEDWT** |  |  |  |  |  |  |  |  |  |  |  |  |
| ≤2SD | 130 (7) | Reference |  | 205 (11) | Reference |  | 212 (12) | Reference |  | 348 (20) | Reference |  |
| >2SD | 31 (13) | 2.6 (1.8-3.8) | **<0.001** | 42 (18) | 2.3 (1.7-3.2) | **<0.001** | 38 (22) | 2.5 (1.7-3.5) | **<0.001** | 48 (27) | 1.9 (1.4-2.6) | **<0.001** |
| **LVEF** |  |  |  |  |  |  |  |  |  |  |  |  |
| ≥-2SD | 150 (7) | Reference |  | 233 (11) | Reference |  | 223 (11) | Reference |  | 358 (18) | Reference |  |
| <-2SD | 11 (13) | 2.4 (1.3-4.4) | **0.006** | 14 (17) | 1.9 (1.1-3.3) | **0.016** | 27 (29) | 3.4 (2.3-5.1) | **<0.001** | 38 (40) | 3.4 (2.4-4.7) | **<0.001** |

MACE = major adverse cardiovascular events: myocardial infarction (MI), resuscitated cardiac arrest, stroke, mortality related to coronary heart disease (CHD), cardiovascular disease (CVD), arteriosclerotic disease or stroke, heart failure; ACE = all cardiovascular events: MACE, angina, peripheral vascular disease (PVD), percutaneous transluminal coronary angioplasty (PTCA), coronary artery bypass grafting (CABG), transient ischemic attack (TIA); HR = hazard ratio; CI = confidence interval; LV = left ventricular; M = mass (g); i = indexed to body surface area (m^2^); EDV = end-diastolic volume (ml); ESV = end-systolic volume (ml); EDD = end-diastolic diameter (mm); EDWT = end-diastolic wall thickness (mm); EF = ejection fraction (%)

^1^ Group defined by the standard deviation threshold: ≤2SD (participants with values ≤ mean + 2*SD), >2SD (participants with a value > mean + 2*SD = upper limit of the normal reference range), ≥-2SD (participants with values ≥ mean - 2*SD), <-2SD (participants with values < mean - 2*SD = lower limit of the normal reference range).

^2^ n = absolute number of events that occurred in the group, % = participants with events of all participants in the group
